# Changes in Serum IL-12 Levels following the Administration of H1-Antihistamines in Patients with Chronic Spontaneous Urticaria

**DOI:** 10.3390/jpm14030295

**Published:** 2024-03-10

**Authors:** Corina Daniela Ene, Milena Tocut, Mircea Tampa, Simona Roxana Georgescu, Clara Matei, Iulia Maria Teodora Leulescu, Ilinca Nicolae, Cosmin Ene

**Affiliations:** 1 Department of Nephrology, ‘Carol Davila’ Nephrology Hospital, 010731 Bucharest, Romania; corina.ene@umfcd.ro; 2 Department of Nephrology, ‘Carol Davila’ University of Medicine and Pharmacy, 020021 Bucharest, Romania; 3 Wolfson Medical Center, Holon 58100, Israel; milena.tocut@gmail.com; 4 School of Medicine, Faculty of Medical and Health Sciences, Tel Aviv 69978, Israel; 5 Department of Dermatology, ‘Carol Davila’ University of Medicine and Pharmacy, 020021 Bucharest, Romania; matei_clara@yahoo.com; 6 Department of Dermatology, ‘Victor Babes’ Clinical Hospital for Infectious Diseases, 030303 Bucharest, Romania; iulialeulescu@gmail.com (I.M.T.L.); drnicolaei@yahoo.ro (I.N.); 7 Department of Urology, ‘Carol Davila’ University of Medicine and Pharmacy, 050474 Bucharest, Romania; cosmin85_ene@yahoo.com; 8 Department of Urology, “Saint John” Clinical Emergency Hospital, 042122 Bucharest, Romania

**Keywords:** chronic urticaria, IL-12p70, IL-12p35, IL-12p40, inflammatory phenotype, anti-H1 therapy

## Abstract

Introduction. Research regarding the role of the IL-12 cytokine family in modulating immune and inflammatory responses is continuously evolving. In this study, the contribution of the p35 and p40 subunits as monomers (noted as IL-12p35 and IL-12p40) and heterodimers (noted as IL-12p70 or IL-12p35/p40) was analysed in the pathophysiology and progression of chronic spontaneous urticaria (CSU). Materials and methods. We conducted a longitudinal, case–control study involving 42 CSU cases and 40 control cases comprising adults without associated conditions. Serial measurements were performed to assess the serum levels of IL-12p70, IL-12p35, and IL-12p40 at the onset of the disease (pre-therapy phase) and 6 weeks after the initiation of the treatment (post-therapy phase). Results. During the pre-therapeutic phase of CSU, elevated serum levels of IL-12 cytokine subtypes were detected compared to the control group. The relationship between IL-12 profiles and the course of CSU highlighted the pro-inflammatory role of IL-12p70 and the anti-inflammatory role of IL-12p35. Significant correlations were observed between IL-12p70 levels and the duration of the disease, as well as between IL-12 and the effectiveness of H1-antihistamines. Conclusions. The molecular background for the pleiotropic activities mediated by IL-12-derived cytokines in patients with CSU lies in the strict regulation of the production, signalling pathways, and cytokine-specific influences on the same pathophysiological events. The results of the present study suggest that the superficial layers of the skin serve as a cellular source of IL-12, a cytokine produced through antigenic stimulation. In patients with CSU, we identified independent, additive, or divergent functions of IL-12p70, IL-12p35, and IL-12p40, all relevant to systemic inflammation. These findings prove that the prototype programming of IL-12 is abnormal in CSU.

## 1. Introduction

Chronic spontaneous urticaria (CSU) is a chronic inflammatory disease with variable clinical characteristics, which can negatively impact the patient’s quality of life [1,2,3,4,5]. The pathophysiology of urticaria is multifactorial, and current therapeutic strategies fail to induce remission in all patients [5,6,7,8,9,10,11].

It is known that urticaria is classified according to the duration of symptoms into two categories, acute (≤6 weeks) and chronic (>6 weeks), as well as according to triggering factors: spontaneous and inducible urticaria. Current evidence shows that the activation of mast cells rich in high-affinity receptors followed by rapid degranulation, the development of autoimmunity (type I, defined by IgE antibodies against autoantigens or type 2b, defined by IgG anti-IgE and IgG anti-FcεRI autoantibodies), and the alteration of cytokine–chemokine networks are intensely debated pathogenetic mechanisms in CSU. It is known that urticarial lesions are induced by vasoactive mediators, with the histamine released by mast cells remaining the main representative process. The genetic polymorphism of mast-cell-derived interleukins and the predisposition to develop CSU are also areas that require study and exploration [12,13,14,15,16,17].

CSU is one of the most common dermatological diseases; however, its pathogenesis is unclear. Autoimmunity and the alteration of the cytokine–chemokine network have received considerable attention lately. There is increasing evidence that CSU is not simply a histamine-mediated disease but an inflammatory disease caused by type 2 immune dysregulation. Numerous inflammatory cells (mast cells, macrophages, basophils, eosinophils) have been detected in the skin lesions of patients with CSU in association with an excessive production of inflammatory mediators (IL-4, IL-13, IL-5, IL-9, IL-10, IL-17, TNFα, IL-31, and IL-33). Systemic inflammation in CSU is supported by significantly elevated serum/plasma levels (compared to healthy individuals) of pathogenic cytokines involved in Th2 inflammation [5,14,16,17]. Many studies have examined the role of T cells and related cytokines in the development of CSU, but there has been limited research focusing on changes in cytokine levels during treatment.

Cytokines play a central role in monitoring and regulating immune and inflammatory responses in allergic processes [2,4,7,18,19,20,21,22,23,24]. The meticulous quantification of cytokines provides valuable information about pathophysiological processes, therapy adjustment, diagnosis, and the stage and prognosis of various human conditions [25,26,27,28,29,30,31,32,33,34,35]. Investigating the involvement of the IL-12 cytokine family in CSU management is a challenge. The IL-12 family consists of five natural members, namely IL-12, IL-23, IL-27, IL-35, and IL-39, and two synthetic members, IL-X and IL-Y [25,26,27,30,35,36,37,38,39,40,41,42,43,44,45,46,47]. The members of the IL-12 family, including their structure, specific receptor nature, signalling pathways, target cells, and biological activities, have been extensively studied (Table 1) [35,36,37,38,39,40,41,42,43,44,45,46,47].

The first member of the IL-12 family, composed of the subunits p35 (IL-12A, IL-12alpha chain) and p40 (IL-12B, IL-12beta chain), is of interest in both biomedical research and clinical practice [48,49,50]. In inflammatory, neoplastic, and autoimmune conditions, the p35 and p40 subunits can function as heterodimers, homodimers, or monomers, producing multiple biological activities [31,37,39,44,45]. The clinical significance of IL-12 in CSU, considering its pro- and anti-inflammatory effects, has not been thoroughly investigated. In this study, we analysed the relationship between the serum status of IL-12 (IL-12p70, IL-12p35, IL-12p40) and clinical characteristics in patients with CSU at the onset of disease (pre-therapy phase) and 6 weeks after the initiation of treatment (post-therapy phase). Our study could provide a new perspective for a comprehensive understanding of the clinical characteristics of CSU, for initiating further research regarding its pathogenesis or for designing therapeutic strategies.

## 2. Materials and Methods

### 2.1. Patient Selection

We conducted a longitudinal case–control study with serial measurements for the systemic quantitative evaluation of IL-12p70, IL-12p35, and IL-12p40 in patients with CSU at the onset of disease (pre-therapy phase) and 6 weeks after (post-therapy phase) versus controls. The patients were treated with H1-antihistamines (in monotherapy or combination) according to disease severity.

The study included 42 patients with CSU and 40 control subjects. The groups were similar regarding their demographic and clinical characteristics. Informed consent was obtained from all the study participants. The study protocol received approval from the Ethics Committee of “Dr. Victor Babes” Hospital (2/15.02.2018). All procedures and experiments conducted in the study adhered to the ethical standards outlined in the Helsinki Declaration.

We enrolled individuals who were not undergoing treatment with antihistamines, corticosteroids, immunosuppressants, or nutritional supplements. The patients were stratified based on CRP (C-reactive protein) levels and UAS7 (Urticaria Activity Score over 7 days) (Table 2).

### 2.2. Laboratory Test

Morning blood samples were collected and subsequently centrifuged at 1500× *g* for 10 min. The resulting serum samples were then preserved at a temperature of −80 °C.

ELISA kits were used to evaluate the quantitative systemic levels of IL-12p70, IL-12p35, and IL-12p40 in the study participants:

1. Human IL-12p70 (Interleukin 12p70, IL12A, and IL12B heterodimer, disulfide-linked) ELISA Kit from ELABSCIENCE, Cat.No.: E-EL-H0150;
2. Human IL-12 p40 (Interleukin 12 p40, IL12B gene in genomic location band 5q33.3) ELISA Kit from ELABSCIENCE, Cat.No.: E-EL-H0151;
3. Human IL-12 p35 (Interleukin 12 p35, IL12A gene in genomic location band 3q25.33) ELISA Kit from Elabscience, Cat.No.: E-EL-H1647.

### 2.3. Statistical Analysis

We used the Kolmogorov–Smirnov test to assess the normality of the data. The comparison between two groups was performed using the non-parametrical Wilcoxon test and the triple comparison of the groups was made using the Kruskal–Wallis test and the Dunn post hoc test. Spearman’s correlation coefficient (rho) was used to evaluate the relationship between pairs of two parameters. We chose a significance level (*p*) of 0.05 (5%) and a confidence interval of 95% for hypothesis testing.

## 3. Results

Table 3 presents the IL-12 levels in CSU patients in the pre-therapy phase/post-therapy phase compared to the control group.

Table 4 presents the IL-12 levels in CSU patients according to disease duration in the pre-therapy phase.

Table 5 and Table 6 summarize the serum levels of IL-12 in CSU patients according to CRP values and UAS7 score, respectively, in the active phase.

Table 7 shows the relationship between IL-12 variants and UAS7 and CRP, respectively, in CSU patients.

## 4. Discussion

CSU is a disturbing allergic condition characterized by episodes of exacerbation and remission, usually triggered by unknown factors. The underlying mechanisms of urticaria are still incompletely elucidated, and involve a complex interplay between immune response, chronic inflammation, oxidative stress, and neuroendocrine factors [5].

It has been established that autoimmune mechanisms and the abnormal function of mast cells and basophils are involved in CSU pathogenesis; however, there are many other mechanisms that are unknown. In CSU lesions, a perivascular infiltrate of various cells is often observed. The cytokine profile in lesions indicates the presence of both Th1 and Th2 cells, resulting in an increased expression of IL-4, IL-5, and IFN-gamma. The cytokines promoting a Th2 profile, such as IL-33 and IL-25 in association with vasoactive agents (e.g., VEGF), are identified in urticaria lesions, but not in healthy skin [51]. Recent data show that patients with CSU are associated with autoimmunity, contact sensitization, and food or drug allergies. In line with this, higher levels of IL-6, IFN-γ, and antithyroid antibodies have been found in CSU patients compared to controls. Moreover, IFN-γ correlated with UAS 7 scores [52].

There are several recent studies that have investigated the role of interleukins in the pathogenesis of CSU. The study conducted by Lin et al. revealed higher serum levels of IL-17, IL-31, and IL-33 in CSU patients compared to controls. A positive correlation was observed between the serum levels of IL-17 and the severity of the disease, as well as between the serum levels of IL-31 and the severity of pruritus. Moreover, elevated IL-33 levels were observed in CSU patients with higher total IgE levels. These findings suggest a potential role of these cytokines in the pathogenesis of CSU [53]. Another study analysed the relationship between IL-17 and CRP levels in patients with CSU and revealed significantly higher levels of both IL-17 and CRP in CSU patients compared to healthy subjects. However, there was no significant correlation between IL-17 and CRP levels, suggesting that increased IL-17 levels may mirror a particular inflammatory pathway in CSU [54].

A recent study found that IL-31 is not directly linked to inflammation or atopic status in CSU; however, its expression is influenced by several factors including disease severity and pruritus [14]. Dobrican et al. analysed the serum levels of IL-25, IL-33, and thymic stromal lymphopoietin in a group of CSU patients and healthy controls. They found higher levels of IL-33 and thymic stromal lymphopoietin in CSU patients compared to controls. There were positive correlations between IL-33 and UAS 7 and DLQI. These findings suggest that IL-33 can be seen as a diagnostic biomarker in CSU, which correlates with disease severity [16].

Our study, which focused on the serum profile of IL-12 (IL-12p70, IL-12p35, IL-12p40) in CSU, revealed complex functions of this cytokine, including its involvement in the pathophysiology and variability of clinical characteristics of CSU, disease duration, recovery from acute/inflammatory phases of the disease, and monitoring of the response to anti-H1 treatment in the patients followed in this study (Table 8). In patients with CSU, the studied IL-12 cytokines exhibited independent, additive, or antagonistic effects in the same pathophysiological context. Therefore, the serum levels of IL-12p70, IL-12p35, and IL-12p40 showed statistically significant variations between CSU patients and the control group, as well as between active and inactive phases of CSU. These variations indicate that IL-12 variants exert independent and convergent functions in the pathophysiology of CSU. Regarding the inflammatory response, IL-12p70 and IL-12p35 exhibited divergent bioactivities, where IL-12p70 promoted inflammation while IL-12p35 inhibited it. The significant reduction in IL-12p70 and IL-12p40 between pre- and post-therapy phases indicates a positive response to anti-H1 treatment (Table 8).

The molecular background for the pleiotropic activities mediated by IL-12 cytokines in patients with chronic spontaneous urticaria (CSU) could be attributed to the strict regulation of production, signalling pathways and their effects, as well as cytokine-specific consequences on the same pathophysiological events. Currently, validated structures and experimental models of the p35 and p40 subunits are available, elucidating the assembly and temporal-spatial organization of polypeptide chains and competition for specific receptors associated with JAK/STAT signalling [43,44,60,61].

In inflammatory conditions, immune cells are primary sources of IL-12 family cytokines in response to the surrounding cellular microenvironment. The independent regulation of the gene expressions of p35 (IL12A gene, location 3q25) and p40 (IL12B gene, location 5q33.3) is crucial. IL12A is constitutively expressed in various cells, while IL12B is expressed only in IL-12p70-producing cells [31]. The production of bioactive IL-12p70 requires the simultaneous expression of both subunits in the same cell, and the heterodimer secretion is limited by the availability of IL12p35 [60,61,62,63,64].

A fundamental contribution to the understanding of the modulation mechanisms of IL-12 family production comes from the β subunit’s ability to regulate the proper folding and assembly of polypeptide chains as a principle in endoplasmic reticulum quality control [62]. In vivo, IL-12α and IL-12β mutually promote each other’s folding, covalent assembly, secretion, and bioactivity [30,43,44]. In the absence of IL-12β, IL-12α misfolds, forming non-native disulfide bonds, and consequently it is retained in cells and subjected to oxidative degradation [62]. The synthesis of free p40 occurs early in response to pathogens [60]. Detected in excess compared to the p70 heterodimer, p40 (as IL12p40 monomer or IL12(p40)2 homodimer) acts as an interface between innate and adaptive immune responses, serving as a natural antagonist for IL-12p70 [7,26]. IL-12p70 is a late product, assembled intra- and extracellularly, requiring specific priming signals from bacterial products and T cell-dependent amplification signals [7,18,30,36,37].

Recent independent studies have validated the concept that IL-12p35 and IL-12p40 are stable cytokines with independent functions, active as monomers, homodimers, and heterodimers, which can have antagonistic, additive, or synergistic influences on the same biological process [43,44]. IL-12-derived cytokines promote a particular phenotype—for example, Th1 (bioactive IL-12), Th2/anti-inflammatory (antagonistic IL-12(p40)2 homodimers)—in a pathogenic microenvironment [61,62,63,64].

IL-12α has anti-inflammatory properties, suppressing lymphocyte proliferation, inducing B cell proliferation, antagonizing pathogenic Th17 responses, and signalling IL-12 [40,43,44,62]. Free IL-12p40 and covalent IL-12(p40)2 homodimers suppress the bioactivity of IL-12p70 and its interaction with IL-12Rβ2 [31]. Additionally, IL-12p40 induces nitric oxide synthase expression and acts as a chemoattractant for macrophages [43,47,63]. IL-12α and IL-12β can associate to form IL-12p70. IL-12p70 is proinflammatory, stimulating inflammation through Th1 differentiation [43,44,62]. On the other hand, it can induce IL-10 expression and act as an anti-inflammatory during secondary responses [42].

The bimodal effects of IL-12-derived cytokines are in line with the idea that IL-12, on the one hand, is a crucial factor driving Th1 responses and IFN-y production in the initial stages of an immune response, and on the other hand, IL-12 may play a later immunoregulatory role in advanced-stage inflammation [64]. Proinflammatory cytokines facilitate the initiation and propagation of autoimmune inflammation and stimulate immune cells, while anti-inflammatory cytokines inhibit inflammation and suppress the immune system [3]. Consequently, a dynamic and ever-changing balance between IL-12p35, IL-12p40, and IL-12p70, with pro- and anti-inflammatory properties, could potentially play a role in the pathogenesis and clinical characteristics of CSU, initiating/regressing inflammation, recovering from acute phases of the disease, and monitoring the anti-H1 response in CSU patients [5,43,44,59,62,63,64].

This research article reveals that CSU is characterized by dysfunction in IL-12 variants, often exacerbated by inflammation. These results need validation in more extensive studies and multiple research centres. Recently, a germline mutation in the ring finger protein 213 gene (RNF213) has been documented, leading to severe urticarial lesions, skin eruptions, and hypercytokinemia [58]. Future studies will aim to understand the relationship and hierarchy between genetic studies, defects in the biogenesis, and dynamics of IL-12 in disease states [58,59].

Our study provides valuable insights into the dynamics of IL-12, as we conducted measurements of serum levels of this interleukin in patients with CSU, both pre- and post-therapy. Our findings enhance the understanding of IL-12 cytokine involvement in CSU progression and treatment responses. The study was conducted at a single centre and included a small number of patients; thus, further multicentre studies involving a larger number of patients are needed to validate the results obtained.

## 5. Conclusions

To summarise, IL-12 cytokines are essential for initiating the protective Th1 response to allergens. In CSU, we observed notable variations in serum IL-12 levels (IL-12p35, IL-12p40, IL-12p70) based on the severity and duration of the disease, the extent of inflammation, and the impact of H1-antihistamines. IL-12p70 promotes inflammation and stimulates immune cells, while IL-12p35 inhibits inflammation and suppresses the immune system. These findings provide evidence that the programming of IL-12 (IL-12p35, IL-12p40, IL-12p70) is involved in the pathophysiology of CSU.

## Figures and Tables

**Table 1:** IL-12 family heterodimeric members.

Members	Quaternary Structure	Receptors	Signalling Pathway	Target Cell
IL-12 (native)	IL-12p35/IL-12p40 (12A/12B, disulfide−linked)	IL-12Rβ1/IL-12Rβ2	JAK/STATs 1,3,4,5	Th_1_ cells
IL-23 (native)	IL-23p19/IL-12p40 (23A/12B disulfide−linked)	IL-12Rβ1/IL-23R	JAK/STATs 1,3,5	Th_17_ cells
IL-27 (native)	IL-27p28/Ebi3 (27A/Ebi3, uncertain−linked)	IL-27Rα/gp130	JAK/STATs 1,4	Tr_1_ cells
IL-35 (native)	IL-12p35/Ebi3 (12A/27B, non−covalent)	IL-12Rβ2/gp130 gp130/gp130 IL-12Rβ2/IL-12Rβ2 IL-12Rβ2/IL-27Rα IL-12Rβ2/WSX-1	JAK/STATs 1,3	Th_17_ cells T_eff_ cells T_regs_ cells B_reg_ cells
IL-39 (native)	IL-23p19/Ebi3 (23A/27B, uncertain−linked)	IL-23R/gp130 gp130/IL-12Rβ1 IL-23R/IL-12Rβ2	JAK/STATs 1,3	B cells
IL-X (synthetic)	Ebi3/IL-23p19 (23A/27B, unexplored−linked)	gp130/IL-23R	JAK/STAT 3	B cells
IL-Y (synthetic)	IL-12p40/IL-27p28 (27A/12B, unexplored−linked)	IL-12Rβ1/IL-27Rα	JAK/STAT 3	T_reg_

p19, p28, p35—α chains; p40, Ebi3—β chains of cytokine heterodimeric complexes; gp130, IL-12Rβ1, IL-12Rβ2, IL-23R, IL-27Rα, WSX-1—subunits of heterodimeric receptor complexes; IL-Interleukin; X and Y—Bioengineered cytokines; Tr-1—type I regulatory T; Th—T helper cells; Teff—T effector cells; Ebi—Epstein–Barr virus; gp130—glycoprotein 130; JAK/STAT—Janus kinase/signal transducer and activator of transcription.

**Table 2:** Patient characteristics.

Patient Characteristics	CSU Group (n = 42)	Control Group (n = 40)	*p* Value
Female/male	29/13	26/14	0.54
Age (years)	31.3 ± 10.8 years	29.8 ± 10.2 years	0.52
BMI (kg/m^2^)	22.1 ± 2.0 kg/m^2^	21.9 ± 2.3 kg/m^2^	0.67
Systolic blood pressure (mmHg) Diastolic blood pressure (mmHg)	120.1 ± 10.6 mmHg 60.0 ± 9 mmHg	110.9 ± 10.0 mmHg 60.1 ± 5 mmHg	0.50 0.53
No of antihistamines (in pre-therapy phase) Patient characteristics	2.66 ± 0.38		
Parameter	CSU patients (pre-therapy phase) (n = 42)	CSU patients (post-therapy phase) (n = 42)	Control group (n = 40)
UAS 7 0 1–6 7–15 16–27 28–42	0 10 (23.8%) 14 (33.3%) 16 (38.1%) 2 (4.8%)	27 (64.2%) 13 (31%) 2 (7.8%) 0 0	-
CRP <0.3 mg/dL 0.3–1.0 mg/dL 1.0–10.0 mg/dL 10.0–50.0 mg/dL	1 (2.4%) 6 (14.3%) 18 (42.8%) 17 (40.5%)	11 (26.2%) 25 (59.5%) 6 (14.3%) 17 (40.5%)	40 (100%)
IgE (UI/mL) <100 >100	26 (61%) 16 (38.1%)	37 (88.1%) 5 (11.9%)	40 (100%)

CSU—chronic spontaneous urticaria; UAS 7—weekly urticaria activity score; urticaria−free = 0; well−controlled urticaria = 1–6; mild urticaria = 7–15; moderate urticaria = 16–27; severe urticaria = 28–42. CRP—C−reactive protein; interpretation according to the EAACI/GA^2^LEN/EDF/WAO guidelines: <0.3 mg/dL—normal level; 0.3–1.0 mg/dL—minor elevation; 1.0–10.0 mg/dL—moderate elevation; 10.0–50.0 mg/dL—marked elevation; >50.0 mg/dL—severe elevation (bacterial infection); n—number of cases.

**Table 3:** IL-12 cytokine levels in CSU patients and controls.

Parameter	CSU Group (n = 42) Pre-Therapy Phase	CSU Group (n = 42) Post-Therapy Phase	Control Group (n = 40)
IL-12p70 (pg/mL)	38.0 ± 14.1	23.6 ± 8.4 p_1_ < 0.001	17.9 ± 4.7 p_2_ < 0.001 p_3_ = 0.007
IL-12p35 (pg/mL)	26.2 ± 10.7	17.3 ± 7.6 p_1_ < 0.005	20.3 ± 3.8 p_2_ < 0.001 p_3_ = 0.012
IL-12p40 (pg/mL)	299.3 ± 89	84.7 ± 26.2 p_1_ < 0.002	77.3 ± 19.2 p_2_ < 0.001 p_3_ = 0.007

CSU—chronic spontaneous urticaria; IL—interleukin; p40 and p35 subunits of IL-12 family; n—number of cases; p—statistical significance; p_0_ < 0.001—triple comparison; p_1_—CSU group (pre-therapy phase) versus CSU group (post-therapy phase); p_2_—CSU group (pre-therapy phase) versus control group; p_3_—CSU group (post-therapy phase) versus control group.

**Table 4:** IL-12 cytokine levels in CSU in the pre-therapy phase according to the period between disease onset and blood sampling.

Parameter	<6 h (G1, n = 9)	6.0–12.0 h (G2, n = 12)	12.0–18.0 h (G3, n = 10)
IL-12p70 (pg/mL)	37.8 ± 12.8	39.0 ± 11.4	37.1 ± 14.2
IL-12p35 (pg/mL)	23.0 ± 8.9	27.11 ± 10.1	26.1 ± 6.2
IL-12p40 (pg/mL)	231.0 ± 32.3	289.5 ± 28.3	307.3 ± 30.6

CSU—chronic spontaneous urticaria; IL—interleukin; p40 and p35 subunits of IL-12 family; n—number of cases; G1 (<6 h); G2 (6.0–12.0 h); G3 (12.0–18.0 h); p_0_ > 0.05—triple comparison.

**Table 5:** IL-12 cytokine levels in CSU in the active phase according to CRP values.

Parameters	0.3–1.0 mg/dL (G1, n = 6)	1.0–10.0 mg/dL (G2, n = 19)	10.0–50.0 mg/dL (G3, n = 17)
IL-12p70 (pg/mL)	28.0 ± 8.1	37.7 ± 10.4 p_1_ < 0.01	43.1 ± 9.7 p_2_ < 0.005 p_3_ = 0.019
IL-12p35 (pg/mL)	28.2. ± 7.7	32.3 ± 9.6 p_1_ = 0.06	16.7 ± 5.8 p_2_ < 0.010 p_3_ < 0.002
IL-12p40 (pg/mL)	209.3 ± 34.7	319.5 ± 36.3 p_1_ < 0.002	308.3 ± 23.6 p_2_ < 0.005 p_3_ = 0.087

CSU—chronic spontaneous urticaria; IL—interleukin; p40 and p35 subunits of IL-12 family; n—number of cases; CRP—C−reactive protein; G1 (0.3—1.0 mg/dL—minor elevation); G2 (1.0—10.0 mg/dL—moderate elevation); G3 (10.0—50.0 mg/dL—marked elevation); p—statistical significance; p_0_ < 0.01 triple comparison; p_1_—G1 versus G2; p_2_—G1 versus G3; p_3_—G2 versus G3.

**Table 6:** IL-12 levels in CSU in active phase according to UAS7 score.

Parameter	1–6 (G1, n = 10)	7–15 (G2, n = 14)	16–27 (G3, n = 16)
IL-12p70 (pg/mL)	32.8 ± 14.1	38.7 ± 16 p_1_ = 0.053	40.9 ± 11.7 p_2_ = 0.044 p_3_ = 0.178
IL-12p35 (pg/mL)	34.9 ± 8.1	29.9 ± 12.1 p_1_ = 0.06	15.8 ± 3.6 p_2_ = 0.010 p_3_ < 0.009
IL-12p40 (pg/mL)	229.3 ± 66.1	338.5 ± 56.8 p_1_ < 0.005	328.3 ± 39.6 p_2_ < 0.005 p_3_ = 0.107

CSU—chronic spontaneous urticaria; IL—interleukin; p40 and p35 subunits of IL—12 family; n—number of cases; UAS7—weekly urticaria activity score; G1 (1–6 well-controlled urticaria); G2 (7–15 mild urticaria); G3 (16–27 moderate urticaria); p—statistical significance; p_0_ < 0.01—triple comparison; p_1_—G1 versus G2; p_2_—G1 versus G3; p_3_—G2 versus G3.

**Table 7:** Correlation between IL-12 variants and UAS7 and CRP, respectively, in CSU patients.

Parameter		IL-12p70	IL-12p35	IL-12p40
IL-12p35	r	−0.64		
	p	0.026		
IL-12p40	r	0.044	0.167	
	p	0.428	0.565	
UAS7	r	0.112	−0.411	0.978
	p	0.015	0.009	0.603
CRP	r	0.411	−0.702	−0.065
	p	0.036	<0.01	0.718

CSU—chronic spontaneous/idiopathic urticaria; IL—interleukin; p40 and p35 subunits of IL-12 family; UAS7—weekly urticaria activity score; CRP—C−reactive protein; p—level of statistical significance; r—coefficient of regression.

**Table 8:** Multifaceted IL-12 (IL-12p70, IL-12p35, IL-12p40) in CSU progression.

IL-12 Involved in CSU	IL-12 Profile	Results of Present Research	Potential Mechanisms
CSU—pathogenesis	The altered production of IL-12 at the onset of the disease and at various stages of CSU (Table 3, Table 4, Table 5 and Table 6). IL-12p35 and IL-12p40 act as independent signalling molecules in CSU (Table 7). IL-12p70 and IL-12p35 may have possibly divergent actions in CSU (Table 7).	Elevated IL-12 in the pre-therapy phase of CSU compared to controls (Table 3). Decrease in IL-12p35 in the pre-therapy phase of CSU versus the post-therapy phase of CSU (Table 4, Table 5 and Table 6).	Recruitment and activation of immune cells infiltrating the skin lesions [7].
CSU—duration	Difference in the synthesis of biologically active IL-12 in CSU (Table 4).	Early IL-12p70, but not p40 or p35 synthesis in CSU (Table 4).	Inflammatory responses arise as an outcome of skin exposure towards harmful stimuli [7,26].
CSU disease activity and response to treatment	IL-12 induces skin injury, including itching, weals/hives, and angioedema (Table 6 and Table 7). IL-12p35 attenuates the progression of CSU (Table 5, Table 6 and Table 7).	UAS7 showed a positive correlation with IL-12p70 and a negative correlation with IL-12p35 (Table 7). Significantly reduced levels of IL-12 between pre- and post-therapy phases (Table 3) indicate a good response to treatment.	Histamine inhibits the production of IL-12 through interactions with H2 receptors [7,55,56,57]. Antihistamines may exert modulatory effects on IL-12-induced T-cell activation. IL-12-induced IFN−γ production was significantly suppressed by cetirizine and fexofenadine [55,56].
Inflammatory profile	Imbalance in IL-12p70/IL-12p35 cytokines (Table 4, Table 5 and Table 7).	Positive correlation between the production of IL-12p70 and CRP, versus a negative association between IL-12p35 and CRP in the active disease (Table 7).	IL-12 amplifies and maintains a Th_1_ phenotype. The IL-12 family orchestrates inflammation through competition between IL-12p70 and IL-12p40 homodimers for binding to the receptor [7,26]. IL-12p35 suppresses inflammation by inhibiting the expansion of pathogenic Th_1_ and Th_17_ cells [40,41,42,43,44].
Outstanding question	Recently, a genetically described disorder has been identified, caused by RNF213 mutation, and is autosomally dominant with complete penetrance. It is characterized by urticarial lesions, eruptions, and hypercytokinemia [58]. Is there a causal role of IL-12 in the susceptibility to CSU?	Future research.	Future studies aim to understand the relationship and hierarchy between genetic studies, alterations in biogenesis, and dynamics of IL-12 in disease states [58,59].

CSU—chronic spontaneous urticaria, IL—interleukin; p40 and p35 subunits of IL-12 family; UAS7—weekly urticaria activity score; CRP—C−reactive protein.

## Data Availability

All data are contained within the article.
